# Adult height prediction using the growth curve comparison method

**DOI:** 10.1371/journal.pone.0281960

**Published:** 2023-02-16

**Authors:** Miha Mlakar, Anton Gradišek, Mitja Luštrek, Gregor Jurak, Maroje Sorić, Bojan Leskošek, Gregor Starc

**Affiliations:** 1 Department of Intelligent Systems, Jožef Stefan Institute, Ljubljana, Slovenia; 2 Faculty of Sport, University of Ljubljana, Ljubljana, Slovenia; 3 Faculty of Kinesiology, University of Zagreb, Zagreb, Croatia; Universiti Malaysia Terengganu, MALAYSIA

## Abstract

Understanding the growth pattern is important in view of child and adolescent development. Due to different tempo of growth and timing of adolescent growth spurt, individuals reach their adult height at different ages. Accurate models to assess the growth involve intrusive radiological methods whereas the predictive models based solely on height data are typically limited to percentiles and therefore rather inaccurate, especially during the onset of puberty. There is a need for more accurate non-invasive methods for height prediction that are easily applicable in the fields of sports and physical education, as well as in endocrinology. We developed a novel method, called Growth Curve Comparison (GCC), for height prediction, based on a large cohort of > 16,000 Slovenian schoolchildren followed yearly from ages 8 to 18. We compared the GCC method to the percentile method, linear regressor, decision tree regressor, and extreme gradient boosting. The GCC method outperformed the predictions of other methods over the entire age span both in boys and girls. The method was incorporated into a publicly available web application. We anticipate our method to be applicable also to other models predicting developmental outcomes of children and adolescents, such as for comparison of any developmental curves of anthropometric as well as fitness data. It can serve as a useful tool for assessment, planning, implementation, and monitoring of somatic and motor development of children and youth.

## Introduction

Height is one of the key indicators of optimal growth, while adult height prediction is of clinical importance in pediatric endocrinology and pediatric exercise science [1]. It is often utilized in children who may be considered for growth hormone therapy due to idiopathic short stature [2], who are investigated for chronic health conditions affecting growth, such as growth hormone deficiency or genetic syndrome [3], in children with diabetes who are experiencing increased growth velocity due to insulin treatment [4], for estimation of maturity status in studies of physical activity, fitness and sport among youth [5, 6], as well in sports talent identification or selection [7, 8].

In routine clinical practice, height is assessed by using normative data based on growth charts and operationalized through percentiles. However, the cross-sectional nature of existing growth curves causes low accuracy in the prediction of final height, particularly during puberty. Cole and Wright [9] recently improved this method by adjusting for the correlation between adult and child percentile, but confidence intervals remained too large for use in everyday clinical and sports practice. In this regard, more sophisticated methods for adult height prediction are needed. The existing approaches for mature height prediction rely either on rather intrusive radiological methods, on nonintrusive anthropometric methods or on a combination of both. The radiological methods assess the ossification of growth plates of hand and wrist. The first model to predict mature height based on a radiological method was presented by Bayley [10], which she later adapted with Pinneau [11], while in 1950 the first radiographic atlas of skeletal development of hand and wrist was published [12]. Since then, several radiological methods have appeared, notably, the Tanner-Whitehouse methods [13] and the Roche-Wainer-Thissen method [14], which rely on manual bone-age estimation, or more recent ones which utilize automated bone age determination [5]. More recently, the Beunen-Malina-Freitas [15] method was developed as a combination of radiological and anthropometric method. All these methods have a common shortcoming of the costliness of assessment of skeletal age and the exposure to radiation with accompanying ethical issues.

In order to develop a nonintrusive and inexpensive method of predicting adult height from anthropometric measurements, the modified Roche-Wainer-Thissen method [16] and the Khamis-Roche method [17] were developed. Both methods estimate adult height from chronological age, child height, weight, and mean height of the parents. Since the height of both parents are frequently not available, the applicability of both methods is limited.

Several mathematical models for predicting adult height from child and adolescent height data alone have been developed as well, but they typically included the timing of peak height velocity (PHV) determining pubertal growth spurt and thus not allowing adult height prediction from pre-pubertal data [18, 19]. In 2005, a method for adult height prediction based on chronological age of an adolescent and measurements of height, sitting height, and weight [20] was developed, but it requires sitting height which is not measured routinely, so the method can only be used on data collected specifically for its purpose.

There have been several attempts to construct linear and non-linear mathematical models for the prediction of growth from height data alone, focusing on the prediction of height growth, but aiming also for the prediction of PHV and other dimensions. All these models are limited by a large number of parameters needed with unclear rationale for their inclusion in the models, and by rather small samples that were used in their construction [13, 21–27]. Probably the most advanced regression models for height prediction were based on Japanese and Korean data [28–30], but this means the models were ethnically specific, and sometimes demanded at least six measurements before the targeted age of prediction. The first attempt to use machine learning (ML) algorithms for the prediction of height, based on adult hand dimensions, was the study by Miguel-Hurtado et al. [31]. Another recent study by Rativa et al. [32] used support vector regression, Gaussian process regression and artificial neural networks, to estimate height and weight from anthropometric measurements. Both aforementioned studies predicted height of the already adult individuals and their results could be used in forensics, textile technology, security and health care, but it wasn’t until recently that the first attempts to use ML for adult height prediction of children from childhood height [33] or parental height [34] were published.

In this paper, we explore the possibilities of using the population data (“big data”) for predicting the mature statue with the help of ML algorithms. The strong advantage of this approach is that the only input required is the height of individuals in a series of consecutive ages, which are typically readily available. The models were trained and evaluated using the Slovenian SLOfit database. The national monitoring system for somatic and motor development of children SLOfit was introduced in 1982 and has been enabling the gathering of annual population data on height, weight, subcutaneous fat and several fitness parameters for generations of children from the beginning of primary school at the age of 6 or 7 to the conclusion of secondary school at age of 18 or 19 [35]. The population database provides a unique opportunity to utilize the cohort data of many thousand individuals in observation of growth patterns and prediction of adult height. Furthermore, the rapid technological development of big data analysis methods enabled us to utilize advanced approaches based on ML to produce the prediction models of children’s development, including the prediction of adult height.

We compare the accuracy of our method, named the Growth Curve Comparison (GCC), with other possible approaches (some of which were used in the related work), namely the percentile method and the methods based on linear regression and decision tree regressors. We further elaborate the performance of the GCC method, which we made publicly available through a web application.

## Materials and methods

The data in our study was obtained from the SLOfit database, the Slovenian national monitoring system of somatic and motor development of school children between 6 and 19 years of age [35]. For our study, we used the data from the 2000–12 SLOfit cohort and extracted a subset of schoolchildren who have recorded all annual height measurements (*i*.*e*. the individual growth curve) during their enrolment in the primary and secondary school, from ages 8 to 18 (we exclude the data at the age of 19 since there is only a small fraction of individuals still enrolled in the system at that age). As the measurements are always conducted in April, we linearly interpolated the height data from two consequent years to the value at the student’s birthday in order to make the data more accurate. By default, the method thus always requires at least two measurements to make a prediction. In total, our subset consisted of data for 7,577 boys and 9,181 girls, totalling 184,338 data points.

The SLOfit study was approved by the Commission of the Republic of Slovenia for Medical Ethics for research (No. 102/03/15). One of the parents or legal guardians provided a written informed consent to include the child in the study.

Since a direct comparison with most of the methods in the related work is not possible–as they use different types of anthropometric data–we focus on the comparison with the approaches that are based on the height time series data, such as we have in our dataset. We compare the performance of the method developed in this study (named the Growth Curve Comparison-GCC) with previously available approaches based on the percentiles (inspired by Cole and Wright [9]) and four machine-learning models based on regression approach (inspired by the ones used in [28–30]). In all cases, the models were independently evaluated for boys and girls.

The *percentile method*, considered as the baseline, assumes that an individual is always in the same rank within the population. Hence, to determine the adult height of the person who is at the N^th^ percentile at a given age, we look at the average height of the said N^th^ percentile at the age of 18.

The next four methods use machine-learning approaches. The *linear regression* method, which is a well-known statistical method, computes the predicted height as a linear combination of the input data (also called features in ML terminology), which are derived from the height values. For height-related features, we considered the following: height at the current year, height at the previous year, growth in the last year and growth in the second-to-last year (*i*.*e*. first derivative), average growth per year up to the current year, maximal growth up to the current year, variance of annual growth values, percentile for the current year, difference between the last year’s growth and the average value for the population, difference between the growths for the last two years, trend of growth per year for last two years (i.e. second derivative), and the distance from the age of 18. Variants of this method use *decision tree regressor*, and *regressor with the* Extreme gradient boosting [36] (*XGB*) algorithm. The age where the peak height velocity (PHV) takes place was used as an additional feature in one set of the XGB models. To estimate that age, we first manually labelled the PHV age for 100 boys and 100 girls, and used this information to fine-tune the model. The features we used were focused on detecting the changes in height growth. When measuring the accuracy, we looked at each year and predicted if PHV happened at this year or not. Our model based on XGB yielded 95% accuracy. The dummy model that predicts for every year that PHV did not happen yielded 83% accuracy (since PHV indeed does not happen in 83% of years).

The *GCC method* takes the growth curve of an individual student up to the chosen year. The method calculates the cosine similarity between the growth curve of this student and those of other students. The cosine similarity between vectors *x* and *y* is defined as [37]

$$k(x,y)=\frac{xy^{T}}{\left||x||||y|\right|}$$

**Fig 1:**
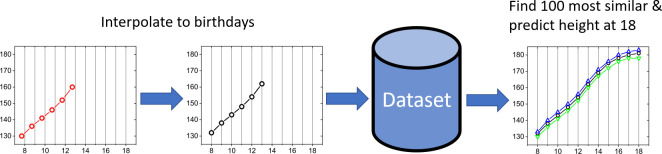
A pipeline showing how a determination of height is made. The input to the model is the time series of heights. We interpolate the values to reflect the heights at the student’s birthday. This set is compared against the database and 100 most similar individuals are then selected, with heights up to the height at the age of 18. The height at the age of 18 for the chosen student is calculated as the sum of the average growths per year. See also Fig 4 for model prediction results.

Based on this metric, the method searches for 100 individuals in the database with the most similar growth curve up to that point, and collects the adult heights for these 100 individuals. Based on these 100 individuals, the method calculates their average growth per year, and sums these growths to make a forecast for a given year. This approach turned out to be more accurate than simply using the median height of the most similar 100 individuals. For illustration, for very tall (or very short) individuals, such an approach would be always wrong as even the most similar individuals are shorter (or taller) than the person whose adult height we are forecasting. The number 100 was chosen as a compromise between accuracy and computational speed, as the reasonable number is somewhere between 75 and 100, while increasing to 150 individuals represents a negligible improvement in accuracy, while demanding a longer computational time. An illustration of the pipeline is shown in Fig 1.

To compare the performance of different models, we first calculated the absolute difference between the actual and predicted height for each student in the testing set, and then averaged these values over all individuals in the set. This metric was calculated for each year, namely using the data from 8-year-olds (a single value) and forecasting height at the age of 18; using the data for 9-year-olds (two values) and forecasting to the age of 18; and all the way to the age of 17. Here, we assume that all students have already reached the adult height at the age of 18 (we commented on that in the discussion section), so no forecast is needed. For the linear regression and regression tree models, this was carried out using a five-fold cross-validation approach. The percentile and the GCC methods were evaluated using all individuals in the set.

## Results

**Fig 2:**
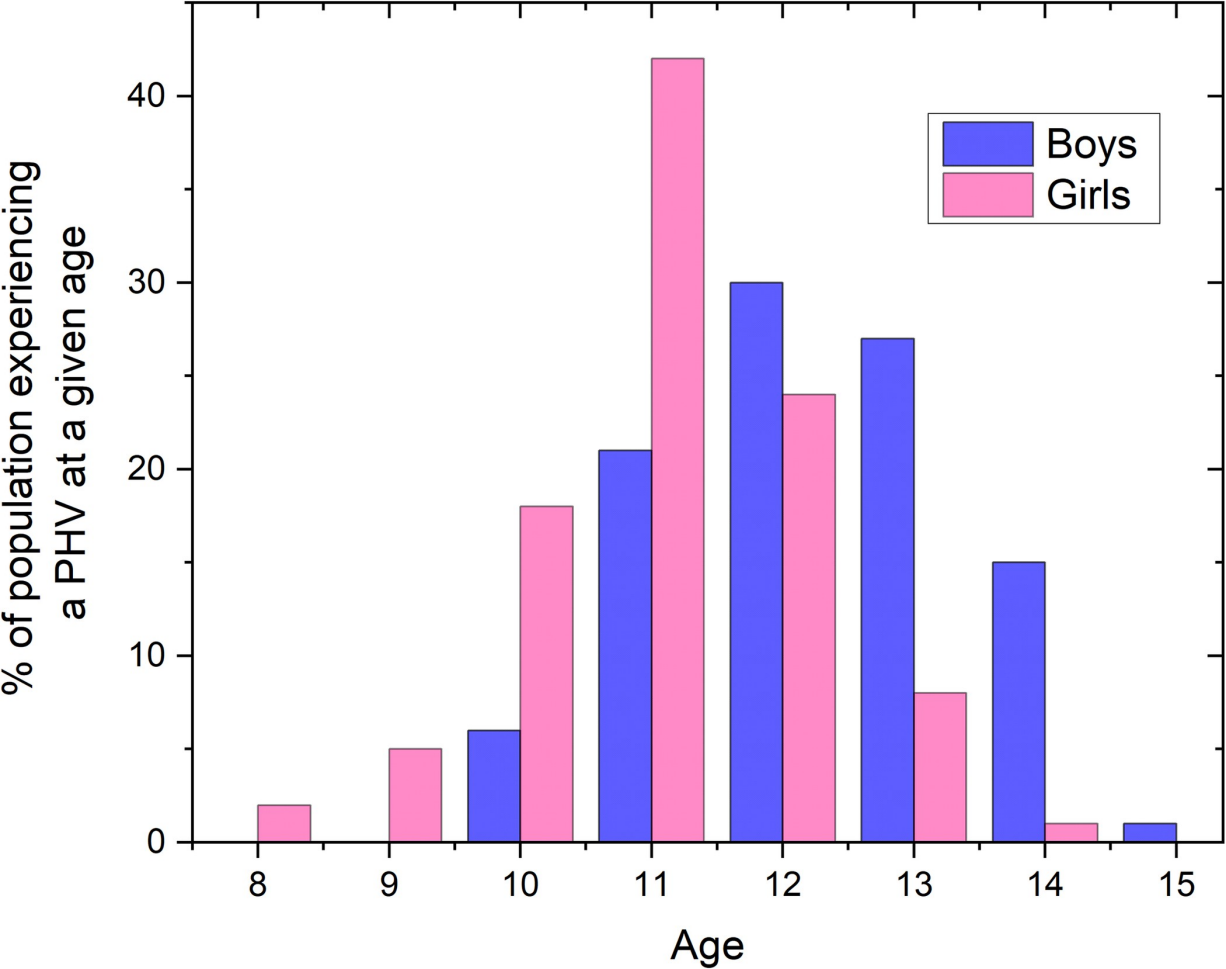
Age at which PHV begins for boys and girls in our dataset.

We first look at our dataset considering the age when PHV begins. The timing of PHV is determined as maximum rate of growth between two annual measurements. Fig 2 shows the distribution for boys and girls (N = 16,758). As expected, for the majority of girls the PHV was between the years 10 and 12, while for boys it was between 11 and 14 years.

**Fig 3:**
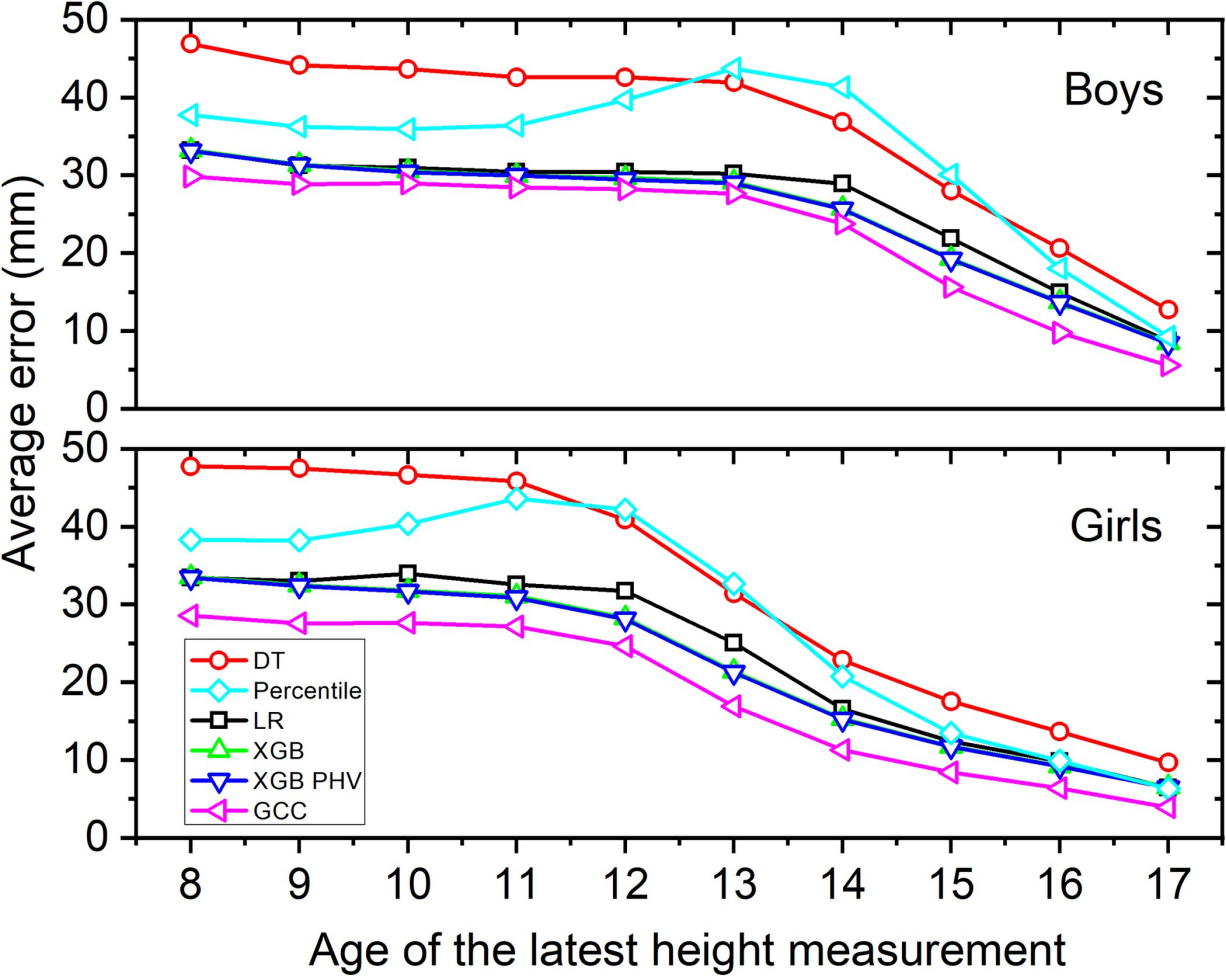
Average error (in mm) for predicting the mature height for boys and girls for six different models (LR = linear regressor, DT = decision tree regressor, XBG = extreme gradient boosting, XGB PHV = XGB using PHV as an additional feature, Percentile = percentile method, GCC = Growth Curve Comparison method), as a function of the year up to which the height data is available.

Fig 3 shows the average error when predicting the mature height (at age 18) for boys and for girls using GCC and five approaches we compare it to. As expected, a general trend for all of them is that the prediction is getting more accurate with the increasing age. For both sexes, the percentile approach shows a substantial increase in error around the age at PHV appears according to Fig 2. The error arises due to the fact that adolescent growth spurt takes place at different times for different individuals, so the model overestimates the mature height in those with an early timing of PHV, and underestimates it in those with a delayed one.

The linear regression model improves the predictions of the percentile method for both sexes over the entire age range. For boys, the average error in determining the mature height is about 3 cm from ages 8 to 14, then the average error sharply drops. The results are similar for girls, with the average error starting to drop after the age of 12, consistent with the earlier onset of puberty. A larger average predicted error in the prepubertal ages is most likely influenced by the fact that pubertal growth is rather independent of prepubertal growth, which means that the accuracy of the prediction is bound to improve after children enter the pubertal growth [38]. The average forecast error at the age of 17 is below 1 cm, where this method is caught by the baseline–as the growth period has generally concluded by that age in most of the population. The decision tree regressor follows this trend but the errors are larger than in linear regression. More complex models than these two can produce more accurate predictions, but can also overfit training data and generalize poorly. The former happened with the more advanced XGB approach, and the latter with the more basic DT. For both sexes, adding the age of PHV as an additional feature does not improve the performance of the XGB approach, which indicates that the PHV is already implied from the other features used in the basic XGB model. The GCC method performs better than the other methods over the entire age span for girls and after the age of 13 for boys. In the following, we analyse the performance of this method in more detail, as it is clearly the best of the used ones to forecast the mature height from the time series of heights.

In the above evaluation, we assumed that we had the height values from the age of 8, but the data may be available only starting at a later age. Tables 1 and 2 show the average error, in millimetres, for the mature height we are forecasting using the heights from age A to age B. For example, the value 23.8 in the first row of Table 1 is the average error for forecasting the mature height for boys using the height values between the ages of 8 and 14.

**Table 1:** Average error (in mm) for forecasting the mature height for boys using the height values beginning at age A and ending at age B.

A↓B→	8	9	10	11	12	13	14	15	16	17
**8**	29.8	28.9	28.4	28.4	28.2	27.6	23.8	15.6	9.8	5.6
**9**		28.9	28.3	28.2	28.0	27.4	23.4	15.5	9.7	5.5
**10**			28.5	28.4	28.0	27.4	23.1	15.2	9.6	5.5
**11**				28.9	28.3	27.8	23.0	15.1	9.5	5.6
**12**					30.7	29.6	23.6	15.1	9.6	5.6
**13**						33.5	27.6	15.3	9.6	5.6
**14**							31.5	16.4	9.7	5.5
**15**								23.3	10.5	5.6
**16**									13.6	5.8
**17**										6.5

**Table 2:** Average error (in mm) for forecasting the mature height for girls using the height values beginning at age A and ending at age B.

A↓B→	8	9	10	11	12	13	14	15	16	17
**8**	28.5	27.6	27.6	27.1	24.6	16.9	11.3	8.4	6.4	3.9
**9**		28.2	27.5	27.3	24.7	16.6	11.2	8.4	6.4	3.9
**10**			29.6	28.5	25.6	16.5	11.2	8.4	6.3	3.8
**11**				31.7	28.4	16.7	11.1	8.3	6.3	3.8
**12**					30.5	17.5	11.0	8.4	6.3	3.9
**13**						24.2	11.3	8.5	6.4	3.8
**14**							15.4	8.8	6.5	3.9
**15**								9.8	6.7	3.9
**16**									6.8	3.9
**17**										4.0

From the two tables we can see that the error is decreasing with the longer series of height values. Again, we see the effect of the adolescent growth spurt on the error–for boys, the largest errors occur when we take short series of heights around the ages of 12 to 14, while for girls this interval is from 11 to 13, when most individuals go through puberty, but at different times. For example, if we look at the first row in Table 2, for girls, we see that the average error changes little until the age of 11 and then drops sharply. We can further see that we still get a similar average error even if we are missing some heights at earlier ages: for example, column B = 13 in Table 2 has almost the same average error regardless of the height time series start at the age of 8 or 11. The GCC approach is feasible even if there are height values at certain ages missing, although the evaluation of all possibilities is beyond the scope of this paper.

**Fig 4:**
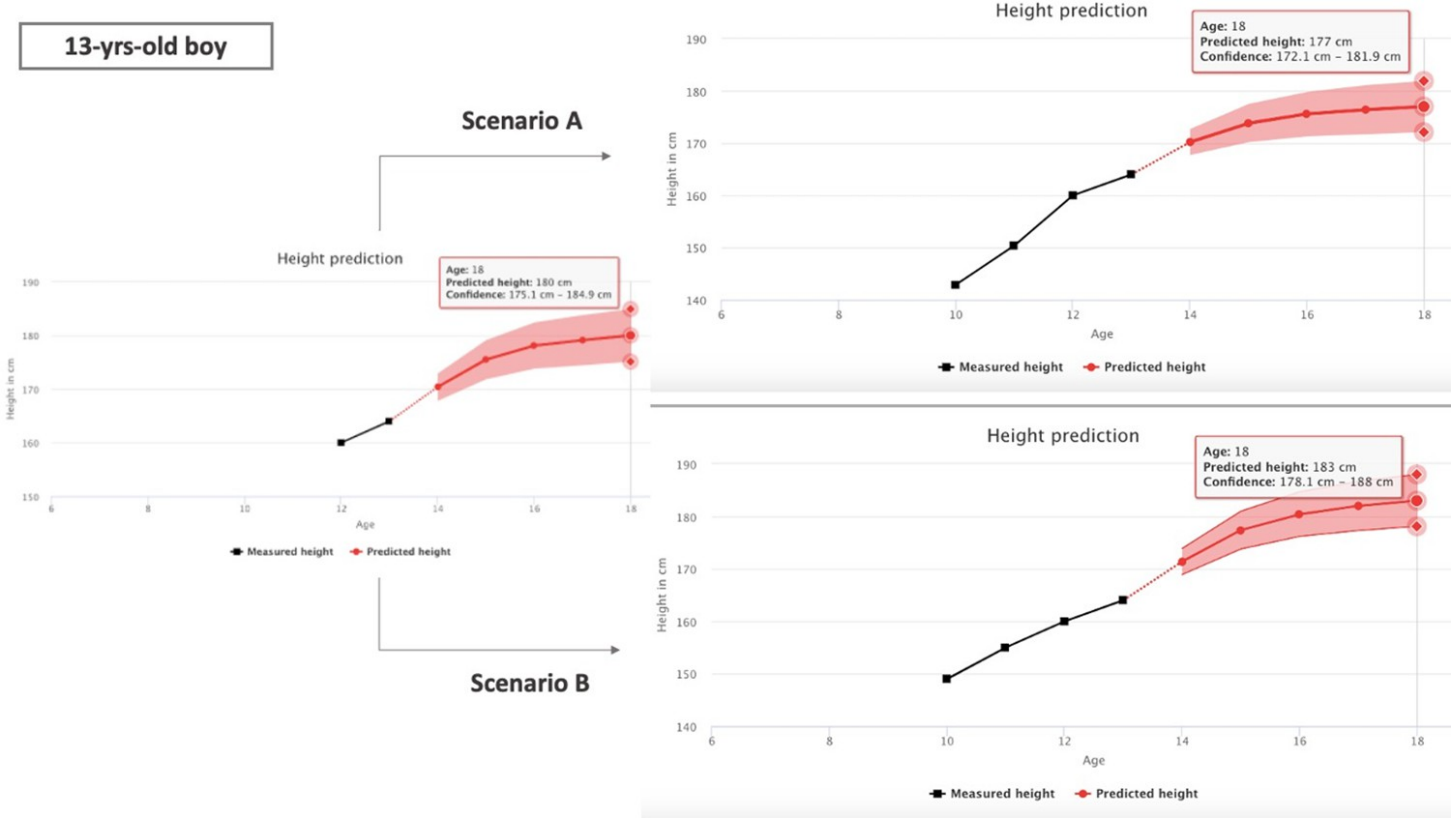
A demonstration of the application performance–measured heights of a 13-year-old boy of average height, together with the height forecast for each specific year by the age of 18, with 95% confidence interval marked. The left panel shows a forecast based on the minimal number of measurements (i.e., two), while the right panel shows 2 forecasts based on a longer time series (i.e., 4 years). Scenario A assumes that the PHV occurred at age 12, while scenario B assumes that the PHV has not yet occurred.

To make the forecasts of the GCC method publicly available, we developed a website application where the user can input a time series of heights to get the year-by-year forecast. The application is accessible at https://en.slofit.org/measurements/height-prediction. Fig 4 shows an example forecast for a 13-year-old boy of average height. The left panel shows that a prediction based on only two measured heights (at age 12 and 13), which does not allow PHV identification, results in the final height of 180 cm. The prediction is improved with the increasing number of data points, which is shown through 2 scenarios for the same 13-year-old in the right panel of the Fig 4. In Scenario A, the PHV has occurred at age 12, which estimates the final height of 177 cm, while in Scenario B, the PHV has not yet occurred which results in the final estimated height of 183 cm. Hence, a forecast in the scenario when the PHV cannot be identified is an approximate average of the predicted final heights in scenarios when the PHV has already occurred and when the PHV is yet to occur.

Tables 3 and 4 list the 95% confidence intervals (CI) (which equals double standard deviation) for predictions using the height values between two ages. The reader should be careful with the interpretation of the values in the tables: while Tables 1 and 2 list the average errors in predicting the mature height using the GCC method, the following tables instead list the intervals for a forecast at a given age C, using the annual height measurements from the age of 8 to the age at the year B. Here, the age of 18 is clearly included as well, as we are forecasting the height at that age. For example, the top left value in the table represents the 95% CI for making a forecast at the age of 10 from heights at the age of 8 and 9 (= A in line with Tables 1 and 2) and indicated with 95% certainty that the true value lies between ± 21.6 mm of the predicted value.

**Table 3:** 95% confidence interval (in mm) for forecasting the height at the age C for boys using the values from the age of 8 to the age B.

B↓C→	10	11	12	13	14	15	16	17	18
**9**	21.6	27.9	39.3	49.2	52.0	49.4	47.1	48.9	50.7
**10**		21.4	35.0	45.2	48.7	47.1	45.7	48.2	50.7
**11**			25.9	39.3	44.7	45.5	45.5	48.3	50.7
**12**				26.6	37.6	42.8	45.4	48.4	50.7
**13**					25.3	36.4	42.9	46.9	49.2
**14**						24.6	35.8	41.7	45.5
**15**							24.3	32.4	37.5
**16**								22.1	29.5
**17**									19.5

**Table 4:** 95% confidence interval (in mm) for forecasting the height at the age C for girls using the values from the age of 8 to the age B.

B↓C→	10	11	12	13	14	15	16	17	18
**9**	24.5	32.7	36.8	38.2	41.4	46.8	50.6	51.9	52.4
**10**		24.6	31.2	35.9	40.7	46.5	50.8	52.1	52.8
**11**			23.5	32.7	40.0	45.9	50.0	51.4	52.3
**12**				23.0	34.4	42.1	47.0	48.2	48.8
**13**					22.6	32.9	39.4	40.4	41.5
**14**						20.4	29.5	31.1	32.0
**15**							20.6	23.5	24.7
**16**								17.4	21.3
**17**									15.6

## Discussion

Adult height prediction allows parents and professionals to predict a child’s adult height, but the use of existing methods for height prediction is often complicated either by their intrusive nature, large number of diverse required measurements, or demanding calculations. From a clinical and economic point of view, a proper prediction of an individual future growth, based on non-intrusive easily obtainable or routine measurements, is preferable. In this regard we developed a ML algorithm, based on growth curve comparison that enables the prediction of adult height only from one previous height measurements and with greater accuracy than the existing non-intrusive methods that have been utilized in other approaches, based on height time series data [9, 28–30]. In order to make the method easily available and applicable in clinical settings and everyday life, we also integrated the prediction method into a web-application that is freely and readily available for users.

The prediction error of our GCC method is consistently decreasing with the longer series of height values and is generally lower in girls than boys, which is reasonable due to their shorter height on average. Similarly, the average error of other studied methods was declining with increasing age and approaching adult height. In general, the largest prediction error in comparison to GCC method was observed in DT method. At age 8 the average error of DT method was 17 mm and 19 mm larger in boys and girls, respectively, but the error at this age was considerably lower than in a comparable study in the preschool children [33].

Interestingly, even the percentile method, widely used as height-for-age curves [39], produced lower prediction error in childhood than the DT method, although the error of the percentile method increased around the period of adolescent growth spurt. Compared to the GCC method, the average prediction error of percentile method was 15 mm larger in 13-year-old boys, and 17 mm larger in 12-year-old girls.

Furthermore, the more sophisticated XGB method produced larger prediction errors than the GCC method because it tended to overfit the training data. Its performance was poorer in girls with average prediction errors being around 5 mm larger than in GCC method throughout the pre-adolescent period. In boys the average prediction error of the XBG method was around 3 mm larger than of the GCC method, but this difference declined towards adult height. Our identified prediction errors of this method were, nevertheless, much smaller than the reported errors in a study utilizing parental height [34], which reported almost 60 mm prediction error.

The classical LR method performed similarly as the XGB method and thus with larger prediction error than the GCC method, although its predictive error increased around the PHV period. In comparison to existing models, utilizing the LR methods, the predictive error of our GCC model was considerably lower than the 4–5 cm error, reported by Cole et al. [9] or Lee et al. [30].

In comparison to other studies on height prediction, the strength of our study lies in its large set of longitudinal cohort data, covering the entire school age, and our use of advanced ML algorithms. In addition, our study included a very recent cohort, representative of current population of children, which ensures its applicability also in the future. The observed CIs of our proposed GCC model are much narrower than in other tested linear methods and enable fair prediction of adult height already from twos pre-existing height values (or values from which the height at a birthday can be calculated). Still, there are several limitations of this study that are worth noting. Our method is applicable for children and adolescents from 8 to 18 years of age but does not take into account that around one half of boys obtain final adult height after the age of 18, which could lead to slight underestimation of adult height. Next, our GCC model was developed on the representative Slovenian sample, but the method itself can easily be adopted for another population if such data is available. Despite an extensive search for open access datasets, we were unsuccessful in obtaining an appropriate dataset that would allow external validation of the newly developed method in a different population of children. To that end, we encourage researchers that curate proprietary longitudinal height datasets to perform further validation studies using the GCC method in diverse populations to test its generalisability. By having the proposed method publicly available in the form of web application, we are creating a practical and easily accessible tool for different profiles of users. Experts in the field of endocrinology can monitor the growth processes in their pediatric patients, sport coaches can find it of assistance in selecting optimal individuals for sport disciplines, and vice versa, while experts from the field of sport, exercise, and physical education can use it to assess and adapt individual training/exercise/learning plans to maturational status of their young athletes. Furthermore, the method is easily translatable to any set of time series data related to anthropometric or motor development. Although our model is outperforming the existing non-intrusive methods of height prediction, it could be further improved with inclusion of additional data or other height-related metrics, which should be a topic of future research.

## Conclusion

We present several approaches for prediction of the adult height from a series of heights at different ages. Using the Slovenian dataset, we developed a new forecasting method based on the big data approach, named the “Growth Curve Comparison” and contrasted it with several well-known non-intrusive methods in wide use, including the percentile approach and the linear regression. The GCC method compares the available growth curve of an individual with all boys or girls in the dataset and then uses the data of the most similar individuals to predict the adult height. This method has proven superior to all other approaches in the current study and was consequently incorporated in a web application which is publicly accessible on the SLOfit website to parents, teachers, trainers, physicians, and other experts, working in the areas related to children development.
